# Nonvolatile Bio-Memristor Fabricated with Egg Albumen Film

**DOI:** 10.1038/srep10022

**Published:** 2015-05-07

**Authors:** Ying-Chih Chen, Hsin-Chieh Yu, Chun-Yuan Huang, Wen-Lin Chung, San-Lein Wu, Yan-Kuin Su

**Affiliations:** 1 Institute of Microelectronics and Advanced Optoelectronic Technology Center, National Cheng Kung University, Tainan 701, Taiwan; 2 Department of Applied Science, National Taitung University, Taitung 950, Taiwan; 3 Department of Electronic Engineering, Cheng Shiu University, Kaohsiung 833, Taiwan; 4 Department of Electrical Engineering, Kun Shan University, Tainan 710, Taiwan

## Abstract

This study demonstrates the fabrication and characterization of chicken egg albumen-based bio-memristors. By introducing egg albumen as an insulator to fabricate memristor devices comprising a metal/insulator/metal sandwich structure, significant bipolar resistive switching behavior can be observed. The 1/f noise characteristics of the albumen devices were measured, and results suggested that their memory behavior results from the formation and rupture of conductive filaments. Oxygen diffusion and electrochemical redox reaction of metal ions under a sufficiently large electric field are the principal physical mechanisms of the formation and rupture of conductive filaments; these mechanisms were observed by analysis of the time-of-flight secondary ion mass spectrometry (TOF-SIMS) and resistance–temperature (*R–T*) measurement results. The switching property of the devices remarkably improved by heat-denaturation of proteins; reliable switching endurance of over 500 cycles accompanied by an on/off current ratio (I_on/off_) of higher than 10^3^ were also observed. Both resistance states could be maintained for a suitably long time (>10^4^ s). Taking the results together, the present study reveals for the first time that chicken egg albumen is a promising material for nonvolatile memory applications.

Organic-based optoelectronic and electronic devices have recently attracted great attention because of their potential applications in lighting systems, flat panel displays, back lighting modules, radio frequency identification tags, flexible sensors, and flexible displays. These organic-based devices possess numerous advantages, such as simple device structure, favorable scalability, potentially low cost, low power consumption, multiple state property, and three-dimensional stacking capability^1^. Nonetheless, the negative effects of toxic organic materials on the environment and human health pose great concerns for the application of these devices. To overcome such problems, researchers and scientists have extensively investigated potential nontoxic biomaterials as substitutes over the last two decades. Biomaterials are biodegradable, bioresorbable, biocompatible, and environment-friendly^2^. More importantly, complicated chemical synthesis is unnecessary to prepare natural biomaterials. Previous reports have proposed several promising biomaterials to prepare organic-based electronic and optoelectronic devices with deoxyribonucleic acid that can serve as excellent electron-blocking layers in both green and blue organic light-emitting diodes. Such materials present promising uses for nonvolatile transistor memory and write-once read-many-times memory applications^3^,^4^,^5^. Several researchers have also attempted to use the tobacco mosaic virus, cysteine, ferritin protein, and enzymes to fabricate memory devices with various novel structures^6^,^7^,^8^. These studies demonstrate that natural biomaterials can effectively simplify the fabrication process and reduce the manufacturing cost of organic memory devices.

A number of protein-based materials have successfully been utilized to fabricate resistive-type memories. For example, the cationic poly-(allylamine hydrochloride) (PAH)/ferritin nanoparticle multi-layer structure, which is prepared via layer-by-layer assembly, was recently demonstrated as a possible biomemory device with reliable switching property. The electrochemical property of redox proteins was identified as the principal cause of reversible resistance switching^9^. The natural silk fibroin protein extracted from the cocoons of *Bombyx mori* (silkworm) has also been used as an active component of transparent bio-memristors. The switching mechanism observed was mostly ascribed to filamentary conduction^10^,^11^. Gold (Au) nanoparticles embedded in silk fibroin protein have been used to improve the switching property^12^. Chen *et al.* further demonstrated the multi-level memory switching characteristics of sericin incorporated with Au nanoparticles^13^. Considering their many benefits, however, most of the aforementioned approaches involve complicated chemical processes, such as extraction or purification, which inevitably increase fabrication costs and method complexity. In this study, we successfully fabricated and characterized nonvolatile resistive switching memory devices by utilizing chicken egg albumen obtained directly from fresh eggs without additional purification or extraction. The proposed devices feature a simpler fabrication process as well as superior switching properties and reliability compared with most previous approaches.

## Results

**Figure 1:**
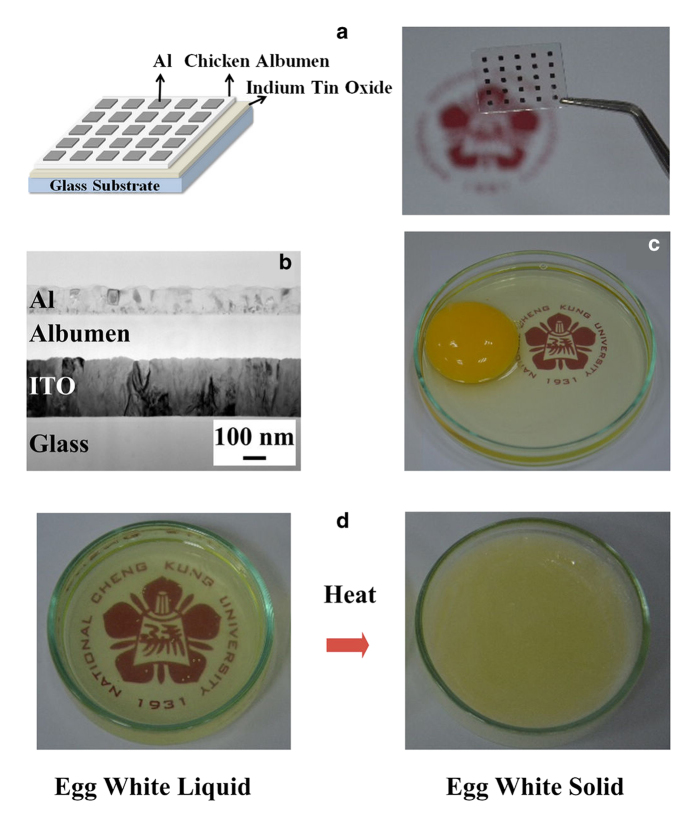
(**a**) Schematic illustration and (**b**) TEM cross-sectional image of the chicken albumen bio-memristor. (**c**) A whole egg consisting of an egg white and an egg yolk. (**d**) Pictures of egg white before and after heating. [The logo in the background were reproduced with permission from National Cheng Kung University]

Figure 1(a) shows a schematic and photographs of an albumen memory on a glass substrate. The device was structured as indium tin oxide (ITO) bottom electrode/albumen insulator/aluminum (Al) top electrode. The albumen thin films were formed by applying a simple spin-coating technique. To understand the switching property of the device, the albumen thin films were treated by two means, including baking at high temperature in an air environment and dry curing at room temperature in a vacuum environment. For convenience, the devices fabricated with thermally baked and dry-cured albumen thin films were called thermal-baked and dry-cured albumen devices, respectively. Fig. 1(b) shows a cross-sectional transmission electron micrograph (TEM) image of the albumen memory devices. Chicken egg is relatively inexpensive and widely available in the market. Egg whites are mostly composed of water (88.5%), protein (10.5%), and carbohydrates (0.5%), as well as minute amounts of other solutions and minerals (0.5%)^14^. Water evaporation, protein–protein interaction, and protein denaturation simultaneously take place when baking at high temperatures. The flexibility and surface hydrophobicity of egg whites also increase with increasing baking temperature^2^,^15^. Fig. 1(c) shows a picture of a chicken egg, while Fig. 1(d) shows the egg white before and after baking.

**Figure 2:**
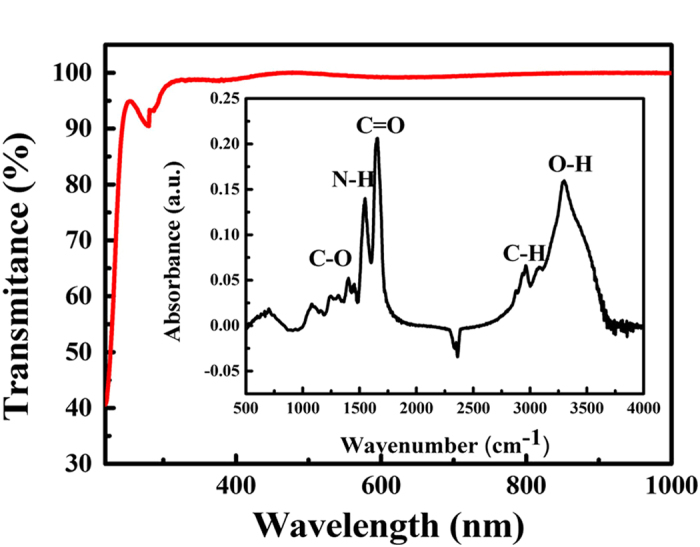
Transmittance spectrum of chicken albumen. Inset: FTIR spectrum of chicken albumen.

Figure 2 shows the transmittance spectrum of the chicken albumen film. The transmittance of the film in the visible wavelength range was between 98.5% and 99.0%, which indicates that the albumen thin film could be considered transparent. The inset in Fig. 2 shows the Fourier-transform infrared (FTIR) spectrum of the chicken albumen film. A significant broad double peak related to hydrocarbon (CH, ~2950 cm^–1^) and hydroxyl (OH, ~3300 cm^–1^) stretching vibrations could be clearly observed from 2500 cm^–1^ to 3700 cm^–1^ 16,17. The peak at 1660 cm^–1^ corresponded to carboxyl (C = O) stretching vibrations^18^, while peaks at 1395 and 1550 cm^–1^ demonstrated carboxylate (C–O) bending vibrations and amide (N–H) bending vibrations, respectively^17^,^19^.

**Figure 3:**
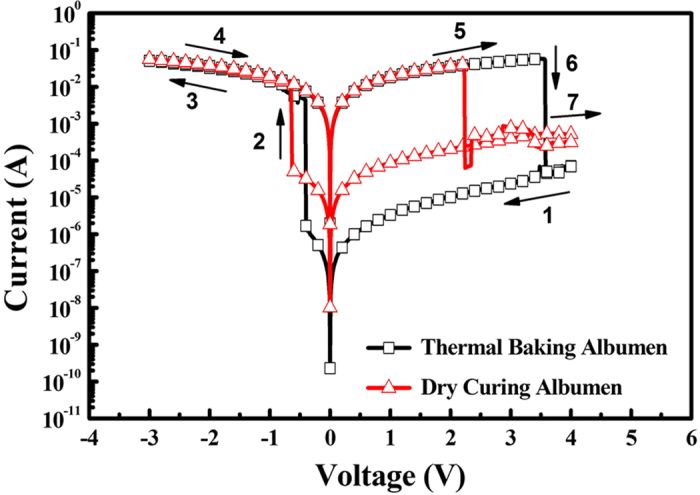
*I–V* characteristics of the thermal-baked and dry-cured albumen devices.

Figure 3 shows the current–voltage (*I–V*) characteristics of the resistive memories fabricated with thermal-baked and dry-cured albumen thin films. The arrows indicate the sweeping direction of the applied voltage. The voltage sweeping rate was set to 1 V·s^–1^. The devices demonstrated a bi-stable resistive switching behavior with an on/off current ratio (*I*_on/off_) larger than 10^3^ at 0.1 V. The reset voltage (*V*_*RESET*_), which refers to a positive critical voltage that induces switching from a low-resistance state (LRS) to a high-resistance state (HRS), of the thermal-baked device was measured to be 3.6 V. The set voltage (*V*_*SET*_), which refers to a negative critical voltage that induces an electrical transition from HRS to LRS, for the same device was measured to be –0.3 V. The *V*_*RESET*_ and *V*_*SET*_ of the dry-cured device were 2.2 and –0.6 V, respectively.

**Figure 4:**
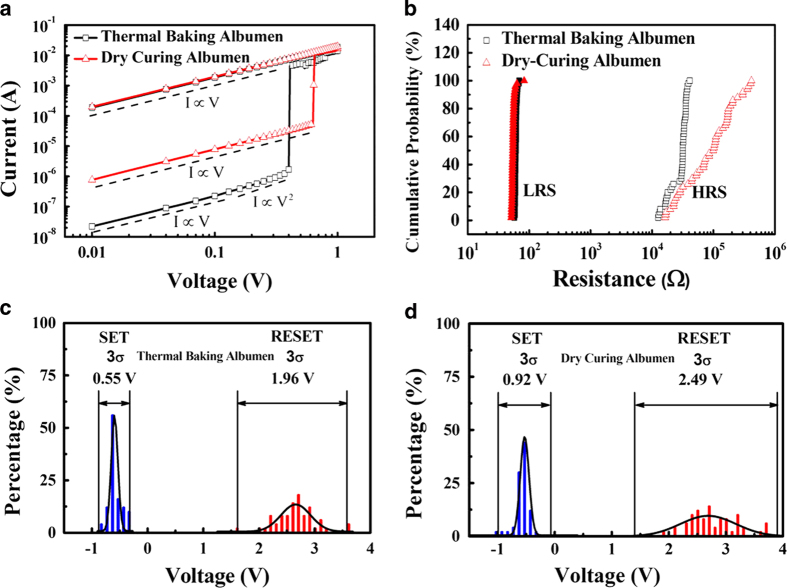
(**a**) Experimental and fitted *I–V* characteristics of chicken albumen in LRS and HRS. The data were plotted in log-log scale. (**b**) *V*_SET_ and *V*_RESET_ distributions of the thermal-baked and dry-cured albumen devices. Cumulative probabilities of the *R*_HRS_ and *R*_LRS_ of (**c**) thermal-baked and (**d**) dry-cured albumen devices.

To understand the current transporting mechanisms further, the reverse *I–V* curves were redrawn in log–log scale, as shown in Fig. 4(a). The logarithmic plot of the *I–V* characteristics of both the thermal-baked and dry-cured albumen devices in LRS could be fitted by a straight line with a slope of 1, which indicates that the conductive current follows Ohm's law. These two devices also showed different types of carrier transportation in HRS. The *I*–*V* curve of the thermal-baked devices was dominated by two complex mechanisms. In the lower applied voltage condition, the slope of the log (*I*)–log (*V*) curve was calculated as 1, which indicates that the conduction mechanism is dominated by ohmic conduction. In the second region, the curve became steeper and the slope was calculated as 2. The observed carrier transportation characteristics are consistent with the trap-controlled space charge limited current (TC-SCLC) model^20^,^21^,^22^. For the dry-cured albumen device, ohmic conduction dominated carrier transportation in HRS. The difference in the fitting results of both devices reveals that the energy required to induce electrical transition from HRS to LRS in the dry-cured albumen device is higher than that in the thermal-baked albumen device.

To analyze the reliability of both devices, their *I–V* characteristics were continually measured for 50 cycles. The resistances of HRS (R_HRS_) and LRS (R_LRS_) as well as the *V*_*SET*_ and *V*_*RESET*_ measurements were acquired from the measurement data and plotted in cumulative probability charts and histograms, respectively. Fig. 4(b) shows the cumulative probabilities of R_HRS_ and R_LRS_. R_LRS_ demonstrated narrow distributions. The median R_LRS_ values for the thermal-baked and dry-cured albumen devices were 54 and 60 Ω, respectively. However, the R_HRS_ distribution of the thermal-baked albumen device was much narrower than that of the dry-cured albumen device. The median R_HRS_ values for the thermal-baked and dry-cured albumen devices were 3.18 × 10^4^ and 9.92 × 10^4^ Ω, respectively. The R_HRS_ and R_LRS_ ratios (R_on/off_) of both devices were larger than 1 × 10^3^. Fig. 4(c,d) show the *V*_*SET*_ and *V*_*RESET*_ distributions of both devices; these distributions were also fitted by using a Gaussian function accompanying three standard deviations (3σ). Differences between the *V*_*SET*_ and *V*_*RESET*_ of both devices were sufficiently large to ensure that the HRS and LRS are clearly distinguishable. However, the *V*_*SET*_ and *V*_*RESET*_ distribution variations of the dry-cured albumen device were obviously larger than those of the thermal-baked albumen device. The average *V*_*SET*_ values of the thermal-baked and dry-cured albumen devices were –0.55 and –0.6 V, respectively, while their average *V*_*RESET*_ values were 2.6 and 2.8 V, respectively. The minimum differences between the *V*_*SET*_ and *V*_*RESET*_ values of the thermal-baked and dry-cured albumen devices were calculated as 1.47 and 2.5 V. The thermal-baked devices demonstrated a narrower distribution of transition voltages and resistance states as well as smaller *V*_*SET*_ and *V*_*RESET*_ values than the dry-cured devices. This result suggests that the signal to noise ratio (S/N ratio) and the discrimination capability of reading memory states could be effectively improved by thermal-baked albumen films.

**Figure 5:**
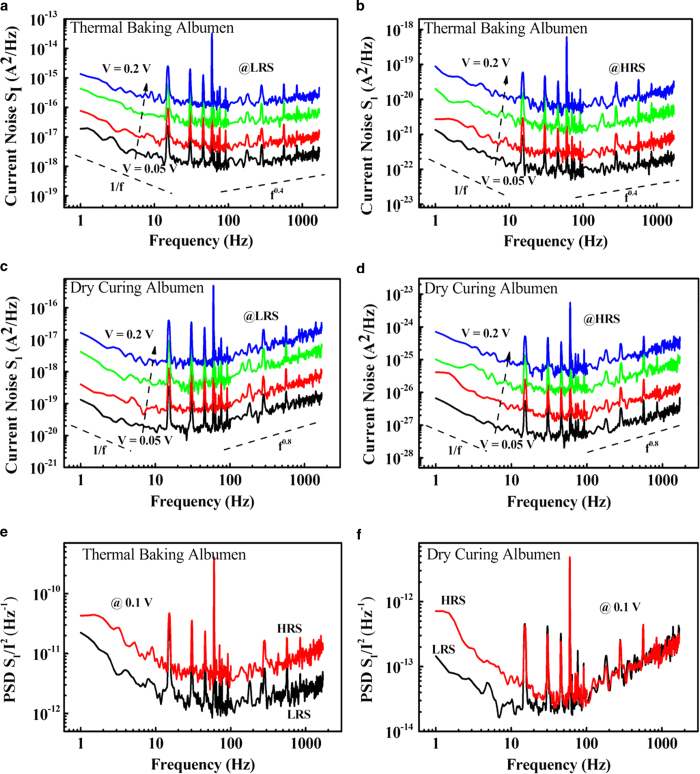
Current noise PSD of the thermal-baked albumen memory as a function of voltage in (**a**) LRS and (**b**) HRS. Current noise PSD of the dry-cured devices as a function of voltage in (**c**) LRS and (**d**) HRS. Noise PSDs of (**e**) thermal-baked and (**f**) dry-cured devices in HRS and LRS with an applied voltage of 0.1 V.

Low-frequency noise (LFN) was also measured to understand the relationship between the resistive switching properties of both devices. Fig. 5(a,c) show the current noise power spectral density (PSD) of the thermal-baked and dry-cured devices as a function of voltage in LRS, respectively, while Fig. 5(b,d) show the PSD of these devices in HRS, respectively. The PSD slopes were close to 1 for both devices, which is attributed to fluctuations in 1/f noise. In the high-frequency region, the current noise PSD spectra of the thermal-baked and dry-cured devices appeared to have similar f^α^ power-law spectra (α > 0). The exponents α for the thermal-baked and dry-cured devices were calculated as 0.4 and 0.8, respectively. The noise PSD was also plotted for both devices in HRS and LRS at an applied voltage of 0.1 V [Fig. 5(e,f)]. The noise PSDs for both devices in HRS were higher than those in LRS. The noise analysis results may be explained as follows. LFN measurement is commonly used to analyze the conduction mechanism, fluctuation sources, and defect status in various electronic devices. Scaling down the dimension of electronic devices could lead to a significant LFN^23^. Among the different noise origins, the 1/f noise is a key concern. The carrier trapping/detrapping phenomenon is generally believed to be the main physical origin of 1/f noise^23^,^24^,^25^. Based on the observed 1/f noise in this experiment, we can conclude that the electron trapping/detrapping phenomenon occurs in albumen thin films and that the current is dominated by electron capture and emission processes in conductive filaments.

In the high-frequency region, the power spectrum for f^α^ power-law noise could be related to the presence of capacitance. A previous report showed that α tends to increase with increasing capacitance^26^. According to the LFN measurement results, the capacitance of the albumen film decreased after thermal baking when compared with that of samples without thermal treatment. After spin-coating, the proteins in albumen were randomly distributed and adhered to the ITO electrode. At relatively high temperatures, the proteins are denatured before they are redistributed and re-adhere to the ITO electrode. This denaturation process could eventually change the capacitance of the resultant albumen thin films^27^,^28^. Considering the noise PSDs of both devices shown in Fig. 5(e) and 5(f), reductions in noise PSD after applying *V*_*SET*_ may be inferred to result from stable current flow in LRS, which could be attributed to the formation of more conductive filaments^25^.

**Figure 6:**
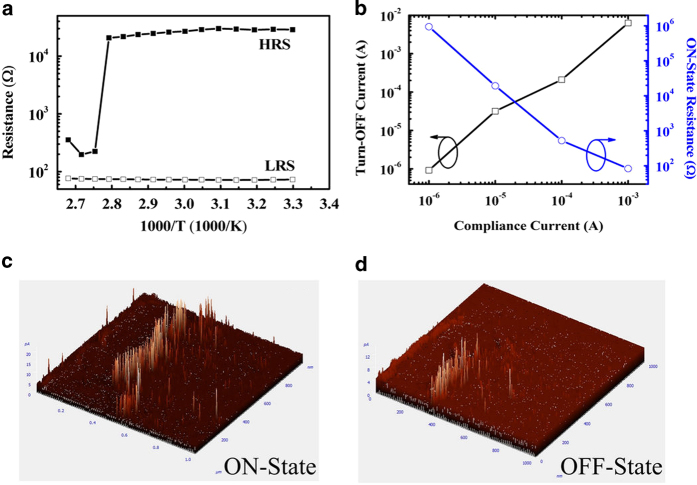
(**a**) Temperature dependence of the resistance of the thermal-baked albumen memory in HRS and LRS. (**b**) Turn-off current and turn-on resistance of the thermal-baked device as a function of switching-on compliance current. (**c**) Local conductivity distribution of the albumen thin film in (**c**) LRS and (**d**) HRS as measured by C-AFM.

The resistances in HRS and LRS at a bias of 0.1 V were recorded at different temperatures, as shown in Fig. 6(a). The resistance of HRS slightly decreased with increasing temperature. However, the resistance abruptly decreased by two orders of magnitude at 357 K. This abrupt drop in resistance may be attributed to intermetallic charge transfer^29^. This specific temperature is defined as the critical transition temperature (*T*_C_). By contrast, the resistance of LRS increased with increasing temperature, and the temperature coefficient of LRS resistance (*β*) could be calculated as 0.001 K^–1^ by fitting the curve of the equation *R*(*T*) = *R*_0_[1 + *β*(*T*–*T*_0_)], where *R*_0_ denotes the resistance at temperature *T*_0_^20^. The turn-on resistance and turn-off current were also measured as functions of the turn-on compliance current, as shown in Fig. 6(b). The turn-on compliance current refers to the compliance current of high-to-low resistance state switching. If the turn-on compliance current is set at a higher value, the amount of induced conductive filaments would increase while the turn-on resistance would decrease. When the device is swept to the OFF-state, the conductive filaments cannot be eliminated completely because the turn-off current tends to increase with increasing compliance current^30^. Moreover, the device in HRS could easily be switched to LRS under a smaller turn-on compliance current because fewer filaments are involved in the current conduction process. The aforementioned variation in turn-on resistance and turn-off current is consistent with basic filament theory. The local conductivity distribution of the albumen thin film was measured by using conductive atomic force microscopy (C-AFM) to verify the filament mechanism. Here, the Pt-coated cantilever tip was used as the top electrode, the scanning area was 100 × 100 nm^2^, and the current distribution topography was measured at a reading voltage of 1 V after writing and erasing operations. Fig. 6(c,d) show that the conductive current paths for LRS are significantly greater than those for HRS. C-AFM measurement results provide clear, straightforward evidence of the conductive filament mechanism of the albumen memory devices. After the traps were filled with injected carriers, the albumen film suffers additional electric field stress and generates filament paths. In general, such electric-field-stress-induced filaments could originate from metal ion diffusion from the electrodes to the insulating film, carbonization associated with localized degradation of the polymer film, or pre-existing oxygen vacancies in the polymer film^20^,^31^,^32^,^33^,^34^,^35^. These filaments could be regarded as the current leakage path leading to different conductivities. From the observed positive temperature coefficient of the LRS resistance, conductive filaments in the albumen film could be confirmed to be mostly composed of metal ions^36^,^37^.

**Figure 7:**
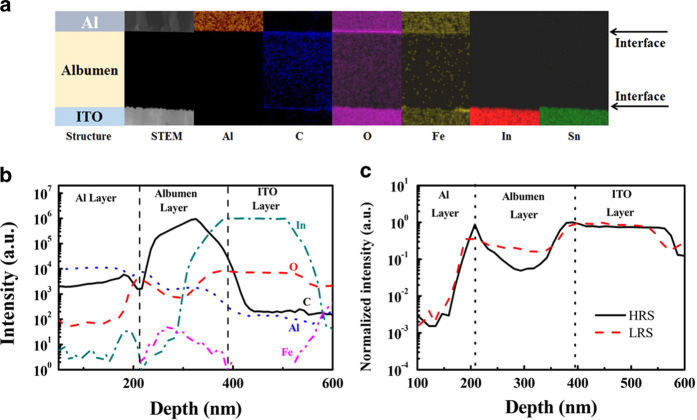
(**a**) STEM-HAADF cross-sectional images of the ITO/albumen/Al memory devices and the EDS mapping images of Al, C, O, Fe, In, and Sn elements. The contrast and brightness of the EDS mapping images are appropriately adjusted for better interpretability. (**b**) Depth profiles of Al, C, In, Fe, and O ions in the albumen memory device as measured by TOF-SIMS analysis. (**c**) Depth profiles of oxygen ions in the thermal-baked albumen memory in LRS and HRS.

Figure 7(a) shows the chemical elements of the ITO/albumen/Al memory devices as determined by high angle annular dark field (HAADF) imaging and energy dispersive X-ray spectroscopic (EDS) mapping analysis in scanning TEM (STEM) mode. Oxygen, iron, and carbon elements were observed in albumen. To verify the compositional variation in chicken albumen film, time-of-flight secondary ion mass spectrometry (TOF-SIMS) was used to profile the ion depth distribution in the memory devices. The depth profiles of aluminum, carbon, indium, iron, and oxygen atoms in the vertical direction of the device are shown in Fig. 7(b). The depth distribution results show that most of the aforementioned atoms within the albumen film in both states present no notable difference, except for oxygen. Fig. 7(c) shows the depth profiles of oxygen atoms in the device after writing and erasing. Here, the SIMS intensity was normalized to the maximum ion intensity of each element. Reductions in the amount of oxygen atoms were clearly observed in the depth range of 225–350 nm. According to the FTIR spectrum in Fig. 2, oxygen functional groups of albumen comprise hydroxyl, carboxyl, and carboxylate groups. The switching phenomenon of polymer memory devices could be ascribed to thin native or electrically oxidized aluminum oxide layers at the Al/polymer interface and formation of filaments^38^,^39^,^40^,^41^. Therefore, we infer that the switching property between LRS and HRS results from the diffusion of oxygen ions between both electrodes.

**Figure 8:**
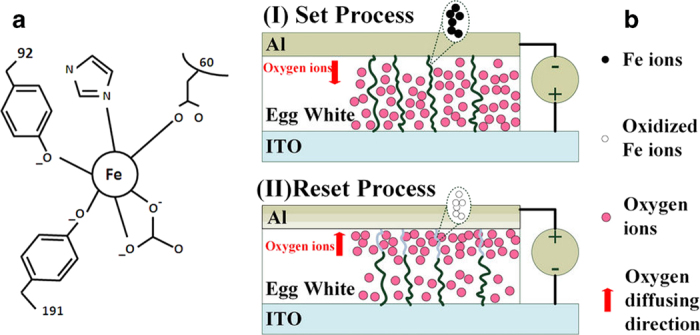
(**a**) Hydrogen-bonding network near Fe binding sites of the N-terminal lobe of ovotransferrin and (**b**) schematic illustration of conductive filament formation and rupture of the albumen-based bio-memristor in LRS and HRS.

Considering the electrochemical property of protein-based materials, direct electron transfer cannot easily take place between the redox-active centers of the protein. Therefore, the surface of the electrodes and transition-metal ions with varying valence from which the redox reaction originates must also be considered^42^. An egg white contains approximately 40 different proteins. The major species of chicken albumen are ovalbumin (54%), conalbumin (12%), ovomucoide (11%), and lysozyme (3.5%)^43^. The egg white also comprises various minerals, such as sodium, potassium, iron (Fe), phosphorus, and fluoride^44^. Among these, Fe ions are the most suitable medium for electron transfer between both electrodes because of the small differences in the work functions between Fe and Al and ITO electrodes. Conalbumin, also known as ovotransferrin, is an iron-binding protein with a high sensitivity to heat^45^,^46^. Fig. 8(a) shows the hydrogen-bonding network near the Fe binding site of the N-terminal lobe of ovotransferrin. The switching mechanism of the albumen-based memory devices may be schematically explained as follows. When a negative voltage is applied, electrons are injected to fill charge trapping defects in the insulator and function as space charges. The albumen film suffers from an enhanced electric field stress. Amino acids are the decomposed products of proteins, and most amino acids are capable of absorbing metal ions or charges. Therefore, we can conclude that the charge trapping effect in egg whites results from the amino acids in protein.^9^ When the magnitude of the applied negative voltage exceeds *V*_SET_, oxidized Fe ions could be reduced and oxygen ions in the cluster of Al atoms would partially escape and diffuse deeper into the albumen film because of the sufficiently large electric field. Conductive filaments for carrier transportation are subsequently formed by Fe ions in the albumen. At this point, the highly resistive albumen film is converted into a low resistance state. After applying a sufficiently large positive voltage *V*_*RESET*_, the reverse process takes place and the filaments are disconnected. Thus, the memory devices recover their high-resistance state. Fig. 8(b) illustrates the filament formation and rupture. The narrower distribution of the transition voltages and resistances of the LRS and HRS as well as the lower average transition voltages for the thermal-baked albumen devices could be attributed to the effects of heat-denatured proteins. Denaturation of proteins could modify the paths of oxygen diffusion and decrease the probability of oxygen scattering, which would enhance opportunities for the formation and rupture of conductive filaments.

**Figure 9:**
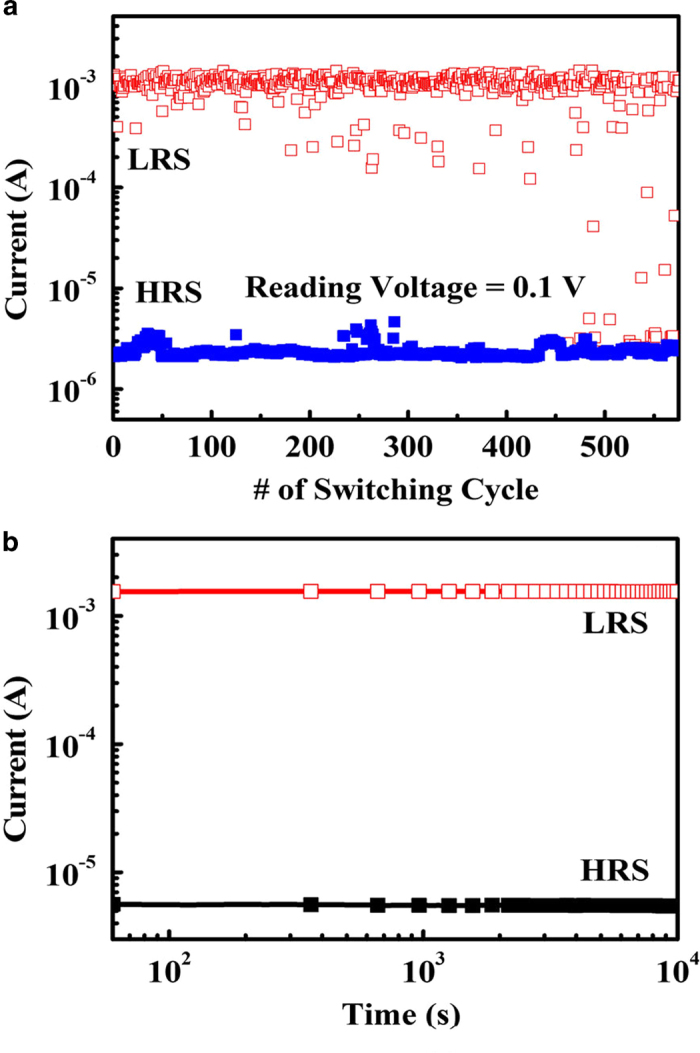
(**a**) Endurance and (**b**) retention properties of the thermal-baked albumen memory. The writing and erasing pulses are set to –1 V/100 ms and 2.5 V/100 ms, respectively, and a reading voltage of 0.1 V is applied.

Figure 9 shows the endurance and retention characteristics of the albumen memory devices. Writing and erasing operations were performed by applying pulse signals of –1 V/100 ms and 2.5 V/100 ms, respectively. The device status after each operation was read with a DC bias of 0.1 V. The measurement results suggest that excellent rewritable characteristics are achieved in the Al/albumen/ITO memory device with a switching cycle of 500 times, despite some minute fluctuations in the repeating ON/OFF state current levels. To evaluate retention performance, the current values of the ON/OFF state were recorded with a reading voltage of 0.1 V. The currents of the devices after writing and erasing operations could be discriminated and sustained for 1 × 10^5^ s, and the *I*_on/off_ was higher than 1 × 10^2^. The retention and endurance characteristics of the albumen memory are thus proven to be reliable and promising for nonvolatile memory applications.

To understand the role and mechanism of albumen in resistive memory, conventional polymeric dielectrics, including poly(methyl methacrylate) (PMMA) and poly (2-hydroxyethyl methacrylate) (PHEMA), were used to fabricate resistive memory devices. Table 1 summarizes the operational characteristics of memory devices with albumen, conventional polymers, and various biomaterials. The albumen memory showed reliable switching properties similar to those devices with conventional polymeric dielectrics. The albumen-based memory also demonstrated superior performance, a simpler fabrication process, and lower fabrication cost when compared with devices comprised of other biomaterials.

In conclusion, we have fabricated and characterized albumen thin-film memory devices. To the best of our knowledge, this study is the first to report this type of devices. Albumen, also known as egg white, is widely available, can be used without complex synthetic procedures such as extraction or purification, and is less expensive than other biomaterials. Electrical bi-stable resistive switching properties were clearly observed in the *I–V* characteristics of the fabricated devices with thermal-baked and dry-cured albumen films. While the current transport mechanisms of both the thermal-baked and dry-cured albumen memory devices in LRS could be described by the traditional Ohm’s law, the TC-SCLC mechanism begins to dominate the system when these devices were switched to HRS. LFN characteristics were also investigated, and filament conduction was proven to be the main mechanism of the albumen devices according to the observed 1/f noise. Membrane capacitance was dominated by the status of the proteins, particularly their redistribution after denaturation by thermal treatment. Compared with dry curing, the redistribution process of proteins during thermal baking could help modify oxygen diffusion paths, decrease the probability of oxygen scattering, and lead to better ON/OFF switching properties. Moreover, the temperature dependence of resistance in both states suggested that the filaments are primarily composed of metal ions. To investigate the role of filaments in device switching between LRS and HRS further, the composition and current distribution variations the of albumen films were respectively analyzed by TOF-SIMS and C-AFM after writing and erasing operations. Filament formation and rupture could be ascribed to electric-field-induced oxygen ion migration and electrochemical redox reaction of ovotransferrin-bound Fe ions. Current transport between cathode and anode occurred because of electron hopping along filaments formed by the Fe ions or redox centers. The albumen memory demonstrated more reliable and reproducible switching behaviors than devices obtained using common polymetric dielectrics, such as PMMA and PHEMA. A long retention time of over 1 × 10^4^ s (about 3 h) was also achieved. These results suggest that bio-memristors with albumen film have great potential applicability in next-generation nonvolatile resistive memory devices. We incorporated CdSe/ZnS quantum dots into the albumen layer and observed improvements in some aspects of its performance. We further aim to fabricate the albumen memory devices on an aluminum foil or a flexible substrate, such as Tetra Pak, for potential application in fresh food packaging. Further research may involve using selenium-rich eggs, which are a popular health food, to fabricate albumen memory devices and determine whether or not excess selenium ions can help improve device performance.

## Methods

### Materials and sample preparation

Chicken eggs were purchased from a local supermarket. The albumen liquid was separated from the egg yolk by using a stainless steel mesh spoon. ITO-coated glass substrates were cut into pieces with an area of 15 × 20 mm^2^ and then cleaned with detergent, deionized water, acetone, and isopropyl alcohol in an ultrasonic bath. The sheet resistance of the ITO film was 10 Ω/sq. The surface of the ITO film on the glass was treated in a UV-ozone cleaner for 25 min.

### Device configuration and fabrication process

The albumen liquid was spin-coated onto the cleaned ITO glass substrate with a spin speed of 4000 rpm for 40 s. The albumen-coated ITO glass substrates were baked at a high temperature in ambient air and dry cured at room temperature in a vacuum environment, respectively. The heating temperature was increased gradually from 100 °C to 120 °C and then to 140 °C and maintained for 10 min at each step. The thickness of the cured albumen film was measured as 270 nm by an Alpha-step surface profilometer. To complete the device structure, a 150 nm-thick aluminum (Al) film was deposited on the device by thermal evaporation through a shadow mask with 1 × 1 mm^2^ square patterns. The Al and ITO films respectively acted as the top and bottom electrodes.

### Device characterization

Electrical characterization of the albumen memory devices was performed by using a high power source meter (Keithley 2400) at room temperature in a glove box filled with nitrogen gas. FTIR was used to investigate the nature of the chicken albumen. UV-Vis spectrophotometry was used to study the optical property of the albumen thin films; here, a blank ITO glass substrate was used for baseline calibration. LFN measurement was used to analyze the switching mechanism of the devices. Cross-sectional TEM images were acquired to examine the structure of the albumen devices. TOF-SIMS was performed to analyze the depth distribution of specific elements in the albumen film after writing and erasing operations.

## Author Contributions

Y.C., H.C., and Y.K. designed this work and wrote the main manuscript. Y.C. and W.L. performed the experiments. Y.C., H.C., C.Y., and W.L. analyzed and discussed the results during the preparation of the manuscript. S.L. contributed to the analysis of noise PSD. All of the authors have reviewed the manuscript.

## Additional Information

**How to cite this article**: Chen, Y.-C. *et al*. Nonvolatile Bio-Memristor Fabricated with Egg Albumen Film. *Sci. Rep.* **5**, 10022; doi: 10.1038/srep10022 (2015).

## Figures and Tables

**Table 1:** Performance characteristics comparison of memory devices with albumen, PMMA, PHEMA, and various biomaterials

**Device**	**Memory Property**			**Ref**
	***I***_***on/off***_	**Retention Time (S)**	**Switching Cycles**	
Thermal-baked Albumen	>10^3^	>10^4^	~ 500	This work
Dry-Cured Albumen	>10^3^	>10^4^	~ 50	This work
PMMA	>10^3^	>10^4^	~ 500	Our previous work
PHEMA	>10^4^	>10^4^	~ 500	20
Silk Fibroin Protein	~ 10	>800	~ 120	10,11
Silk Protein/Au Nanoparticles Blends	>10^6^	>10^2^	>10	12
Enzyme Multilayers	>10^2^	>10^4^	~ 200	8
PAH/Ferritin Nanoparticle Multi-Layers	>10^3^	>10^4^	~ 300	9
Sericin	~ 10^6^	>10^3^	~ 21	13
